# A novel nomogram for predicting respiratory adverse events during transport after interventional cardiac catheterization in children

**DOI:** 10.3389/fped.2022.1044791

**Published:** 2022-10-20

**Authors:** Chaoyang Tong, Peiwen Liu, Kan Zhang, Ting Liu, Jijian Zheng

**Affiliations:** Department of Anesthesiology, Shanghai Children’s Medical Center, School of Medicine and National Children’s Medical Center, Shanghai Jiao Tong University, Shanghai, China

**Keywords:** respiratory adverse events, children, cardiac catheterization, nomogram, predictors

## Abstract

**Objective:**

The rate and predictors of respiratory adverse events (RAEs) during transport discharged from operating room after interventional cardiac catheterization in children remain unclear. This study aimed to investigate the incidence and predictors, and to construct a nomogram for predicting RAEs during transport in this pediatric surgical treatment.

**Methods:**

This prospective cohort study enrolled 290 consecutive pediatric patients who underwent ventricular septal defects (VSD), atrial septal defects (ASD), and patent ductus arteriosus (PDA) between February 2019 and December 2020. Independent predictors were used to develop a nomogram, and a bootstrap resampling approach was used to conduct internal validation. Composite RAEs were defined as the occurrence of at least 1 complication regarding laryngospasm, bronchospasm, apnea, severe cough, airway secretions, airway obstruction, and oxygen desaturation.

**Results:**

The rate of RAEs during transport was 23.1% (67 out of 290). Multivariate analysis identified age (vs. ≤3 years, adjusted odds ratio (aOR) = 0.507, 95% conﬁdence interval (CI), 0.268–0.958, *P* = 0.036), preoperative upper respiratory tract infections (URI, aOR = 2.335, 95% CI, 1.223–4.460, *P* = 0.01), type of surgery (vs. VSD, for ASD, aOR =  2.856, 95% CI, 1.272–6.411, *P* = 0.011; for PDA, aOR = 5.518, 95% CI, 2.425–12.553, *P* < 0.001), morphine equivalent (vs. ≤0.153 mg/kg, aOR = 2.904, 95% CI, 1.371–6.150, *P* = 0.005), atropine usage (aOR = 0.463, 95% CI, 0.244–0.879, *P* = 0.019), and RAEs during extubation to transport (aOR = 5.004, 95% CI, 2.633–9.511, *P* < 0.001) as independent predictors of RAEs during transport. These six candidate predictors were used to develop a nomogram, which showed a C-statistic value of 0.809 and good calibration (*P* = 0.844). Internal validation revealed similarly good discrimination (C-statistic, 0.782; 95% CI, 0.726–0.837) and calibration. Decision curve analysis (DCA) also demonstrated the clinical usefulness of the nomogram.

**Conclusion:**

The high rate of RAEs during transport reminds us of the need for more medical care and attention. The proposed nomogram can reliably identify pediatric patients at high risk of RAEs during transport and guide clinicians to make proper transport plans. Our findings have important and meaningful implications for RAEs risk prediction, clinical intervention and healthcare quality control.

## Introduction

Respiratory adverse events (RAEs) are the most common complication during pediatric anesthesia, characterized by both minor adverse events (oxygen desaturation, airway obstruction or secretions and cough) and major adverse events (laryngospasm, bronchospasm and apnea), with a reported prevalence of up to 50% (1–3). Despite the improvement of existing guidelines for pediatric anesthesia management, RAEs remain one of the leading causes of morbidity and mortality and bring varying levels of physical and psychological trauma to children and parents (4, 5).

Many factors correlated with children's medical history, anesthesia management, and type of surgery contribute to the high rate of this occurrence, and the underlying mechanisms include anatomic and physiological considerations, as well as frequent upper respiratory tract infections (URIs) and inflammation (6–10). Although previous studies have identified several predictors for RAEs during perioperative period, rapid and accurate preoperative assessment of high-risk children by pediatric anesthetists remains a great challenge in clinical practice (11, 12).

Children with congenital heart disease (CHD) are more susceptible to develop viral respiratory tract infections that can cause concomitant cardiac and respiratory compromise, increasing the risk of postoperative respiratory complications (6, 13–15). With the development of interventional technology, CHD is increasingly treated by cardiac catheterization, and the rate of life-threatening events is greatly reduced compared to open heart surgery (16, 17). Nevertheless, the high rate of RAEs remains an unavoidable and intractable problem in this surgical procedure, which may lead to transient damage evolving into unpredictable serious consequences if not treated promptly and effectively (18, 19).

Published studies concerning RAEs mainly focused on the period of anesthesia induction, intraoperative and post-anesthesia care unit (PACU), with relatively abundant medical resources (3, 6–10). However, little attention has been paid to the rate of RAEs during transport discharged from operating room after interventional cardiac catheterization in many pediatric anesthesia practice, which often lacks adequate resources for anesthesia care and monitoring. Further, no prediction model for RAEs during transport after this surgery presenting for anesthesia was established. Thus, this study aimed to investigate the incidence and predictors, and to construct a nomogram for predicting RAEs during transport in this pediatric surgical treatment.

## Materials and methods

### Study design and ethics

This single-center prospective study was approved by the Institutional Review Board (IRB) of Shanghai Children's Medical Center (SCMCIRB-K20170122) and written informed consent was obtained from parents or the legal guardians of each child before surgery. This trial was registered before patient enrollment at the Chinese Clinical Trial Registry (ChiCTR-RRC-17012519). This study was conducted in accordance with the Guidelines of the International Conference on Harmonization of Clinical Norms and the Declaration of Helsinki, and was adhered to STROBE guidelines.

### Patient enrollment

**Figure 1:**
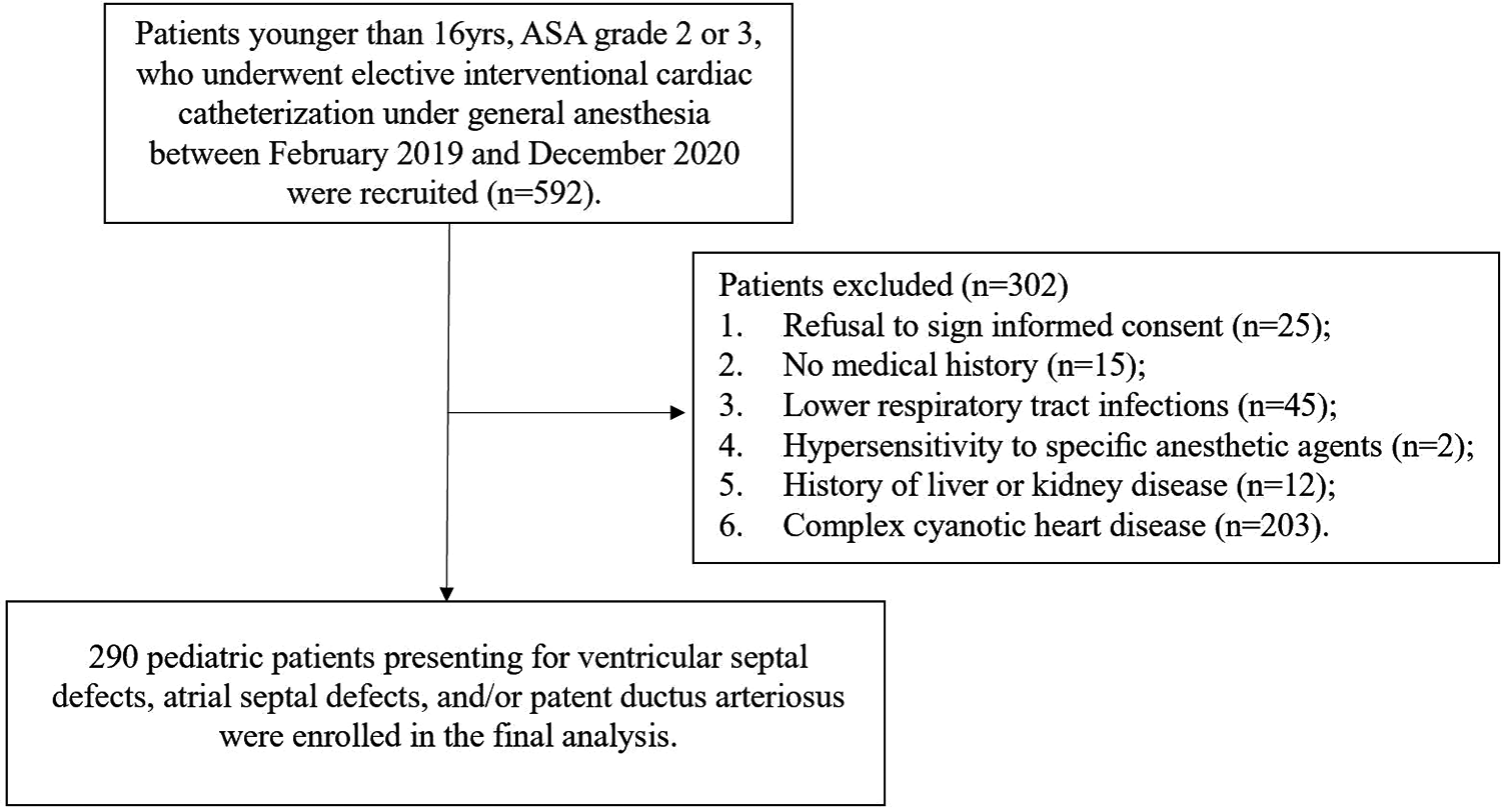
Patient flowchart.

Eligible patients ≤16 years, ASA grade 2 or 3, scheduled for elective interventional cardiac catheterization under general anesthesia (GA) for ventricular septal defects (VSD), atrial septal defects (ASD), and/or patent ductus arteriosus (PDA) from February 2019 to December 2020. Exclusion criteria included parental refusal to sign informed consent, evidence of lower respiratory tract infections (such as pneumonia and bronchitis) within the previous 2 weeks, no medical history (parents or legal guardians could not recall clearly), known hypersensitivity to speciﬁc anesthetic agents, history of liver, kidney disease or complex cyanotic heart disease, and recent participation in other studies. 290 children were enrolled in the final analysis (Figure 1).

### Anesthesia protocols

To minimize other potential bias, all surgical procedures in this study were handled by the same group of surgeons and anesthesiologists. The LMAs were removed by the chief anesthesiologist at the end of surgery when the end-tidal sevoﬂurane concentration dropped below 1% and the respirations became regular. The Aldrete score was the standard reference for discharging catheterization room. When the scores were ≥6, the chief anesthesiologist will consider transferring the pediatric patients. During transport, all pediatric patients were routinely monitored by electrocardiogram and pulse oximetry, and underwent mask ventilation. Additionally, all emergency airway equipment and first aid medicines were fully prepared. Detailed anesthesia protocols were reviewed in our previously published literature (18, 19). Considering the usage for analgesia with fentanyl and sufentanil in all children, we standardized the doses with following method: the total dose in milligrams for each opioid was multiplied by its standard equianalgesic conversion ratio and divided by lean body weight (20–22).

### Data collection, outcomes and definition

Before surgical procedure, the questionnaire form concerning children's demographic information was completed by parents or legal guardians. Intraoperative clinical data and outcomes including emergence agitation, vomiting, fever, and respiratory adverse events (RAEs) were recorded by senior resident anesthetists. Children with any two of the following URI symptoms confirmed by parents or legal guardians within the past 8 weeks were considered to have a history of URI: nasal congestion, runny nose, dry or wet cough, sore throat, sneezing, or fever >38°C (6, 7). Composite RAEs were defined as the occurrence of at least 1 complication including laryngospasm, bronchospasm, apnea, severe cough, airway secretions or obstruction, and oxygen desaturation (6, 7, 23). In this study, the perioperative period was further divided into the following 6 parts, namely, anesthesia induction, intraoperative period, after surgery to extubation, extubation to transport, during transport, and in ward, so as to more accurately study the occurrence of RAEs in different periods.

### Statistical analysis

Statistical power calculations were not performed prior to this study since the sample size was based on available data. Statistics and data analysis plans were defined before accessing the data and were completed after the data were accessed. Continuous variables were compared using Two independent sample *t*-test or Mann-Whitney *U* test based on the rate of RAEs during transport. Categorical variables were compared with Chi-square test or Fisher exact test, depending on the sample size. Univariate analysis showed that all factors significantly correlated with RAEs during transport (*P* < 0.2) were inserted into the multivariate logistic regression model using the forward selection strategy.

The predictive model was presented with a nomogram to provide a visual point system to estimate the probability of RAEs during transport. Hosmer-Lemeshow (H-L) goodness-of-fit test was used to evaluate the model's fit. Discrimination (C-statistic) and calibration (calibration curve) were used to assess the performance of the prediction model. The area under the receiver operating characteristic curve (AUROC) was calculated to reflect model's discrimination. To reduce overfitting and quantify optimism, the nomogram was internally validated with an approach to 1,000 bootstrapped resampling and calculating an optimism-corrected C-statistic. Decision curve analysis (DCA) was used to describe the clinical validity and net benefit of the nomogram (24). Statistical analysis was performed using the SPSS 26.0 software (IBM Corp., Armonk, NY, USA). R version 4.1.2 was used with the packages of rms, tidyr, dplyr, rmda, forestplot, pROC. *P*-value <0.05 was considered statistically significant.

## Results

### Study cohort

From February 2019 to December 2020, 290 pediatric patients underwent this surgical procedure, of which 33.1% (96 out of 290) received ASD, 37.6% (109 out of 290) received VSD, and 29.3% (85 out of 290) received PDA. Also, among all enrolled patients, 34.1% (99 out of 290) and 23.1% (67 out of 290) patients occurred RAEs during extubation to transport and transport discharged from operating room, respectively (Supplementary Figure S1). The most common RAEs was desaturation, found in 127 times (51.0%), followed by airway obstruction in 97 (39.0%), airway secretion in 12 (4.8%) and laryngospasm in 10 (4.0%), and other RAEs including laryngospasm, apnea, and severe cough was uncommon (Supplementary Figure S2).

### Model development

**Figure 2:**
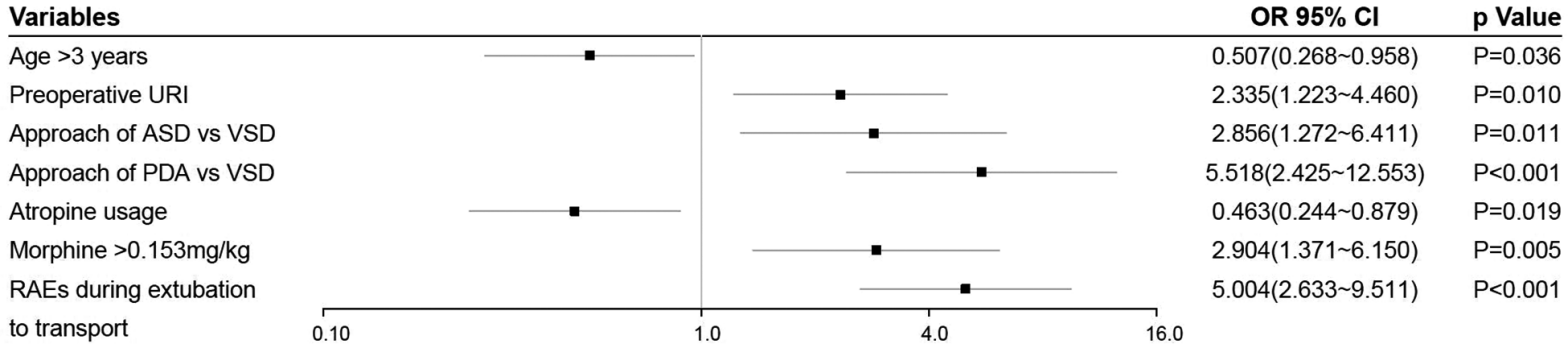
Forest plot of independent predictors of RAEs during transport. URI, upper respiratory tract infection; ASD, atrial septal defects; VSD, ventricular septal defects; PDA, patent ductus arteriosus; RAEs, respiratory adverse events.

**Figure 3:**
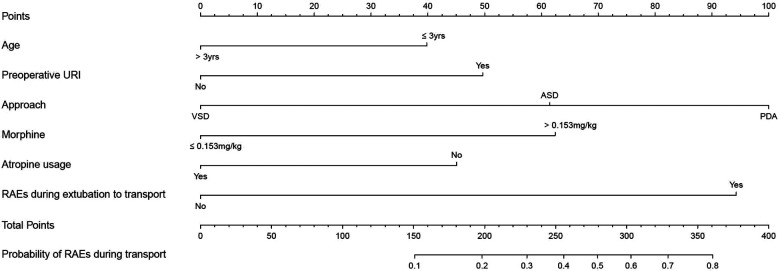
A novel nomogram to predict RAEs during transport. The nomogram provides a visual point system based on the combination of patient characteristics (age, preoperative URI, type of surgery, morphine equivalent, atropine usage, and RAEs during extubation to transport) to estimate the probability of RAEs during transport. To calculate the probability of RAEs during transport, the points of six variables determined on the scale were added to obtain the total points. Draw a vertical line from the total points scale to the last axis to obtain the corresponding probability of RAEs during transport. For example, if a pediatric patient ≤3 years (40 scores), combined with preoperative URIs (50 scores), presenting for ASD (60 scores), accompanied with morphine equivalent >0.153 mg/kg (60 scores) and atropine usage (45 scores), and developed RAEs during extubation to transport (95 scores), the total points were 350 scores, and the corresponding occurrence of RAEs during transport were nearly 80%. URI, upper respiratory tract infection; ASD, atrial septal defects; VSD, ventricular septal defects; PDA, patent ductus arteriosus; RAEs, respiratory adverse events.

Univariate analysis found that seven variables were significantly associated with RAEs during transport (Table 1). Multivariate analysis identified age (vs. ≤3 years, adjusted odds ratio (aOR) = 0.507, 95% conﬁdence interval (CI), 0.268–0.958, *P* = 0.036), preoperative URI (aOR = 2.335, 95% CI, 1.223–4.460, *P* = 0.01), type of surgery (vs. VSD, for ASD, aOR =  2.856, 95% CI, 1.272–6.411, *P* = 0.011; for PDA, aOR = 5.518, 95% CI, 2.425–12.553, *P* < 0.001), morphine equivalent (vs. ≤0.153 mg/kg, aOR = 2.904, 95% CI, 1.371–6.150, *P* = 0.005), atropine usage (aOR = 0.463, 95% CI, 0.244–0.879, *P* = 0.019), and RAEs during extubation to transport (aOR = 5.004, 95% CI, 2.633–9.511, *P* < 0.001) as independent predictors of RAEs during transport (Figure 2). To determine the threshold for morphine equivalent, ROC analysis was performed, which showed the optimal cutoff value was 0.153. Using these six parameters, this study developed a nomogram to predict the probability of RAEs during transport (Figure 3).

**Table 1:** Perioperative characteristics stratified by RAEs during transport.

Variables^a^	RAEs (*n* = 67)	No-RAEs (*n* = 223)	*P* value
Age, years	3.2 ± 2.3	3.8 ± 2.3	0.091
Age >3 years	26 (38.8)	121 (54.3)	0.027*
Sex			0.464
Male	24 (35.8)	91 (40.8)	
Female	43 (64.2)	132 (59.2)	
ASA grade			0.907
II	57 (85.1)	191 (85.7)	
III	10 (14.9)	32 (14.3)	
Height, cm	94.6 ± 17.6	101.2 ± 17.7	0.026*
Weight, kg	14.8 ± 6.1	16.5 ± 7.4	0.080
BMI, kg/m^2^	16.0 ± 1.8	15.8 ± 2.0	0.596
History of allergy	14 (20.9)	30 (13.5)	0.136
History of asthma	1 (1.5)	3 (1.3)	>0.999
History of hay fever	5 (7.5)	22 (9.9)	0.553
Bronchial hyperreactivity	2 (3.0)	4 (1.8)	0.625
Osas	24 (35.8)	70 (31.4)	0.497
Passive smoking	23 (34.3)	65 (29.1)	0.419
Type of surgery			0.006*
VSD	16 (23.9)	93 (41.7)	
ASD	22 (32.8)	74 (33.2)	
PDA	29 (43.3)	56 (25.1)	
Preoperative URI	41 (61.2)	92 (41.3)	0.004*
Propofol, mg	56.3 ± 26.5	61.1 ± 25.8	0.194
Morphine equivalent, mg	0.18 ± 0.04	0.17 ± 0.04	0.189
Morphine equivalent >0.153 mg/kg	53 (79.1)	147 (65.0)	0.041*
Muscle relaxant	1 (1.5)	5 (2.2)	>0.999
Atropine usage	28 (41.8)	139 (62.3)	0.003*
Dexmedetomidine	49 (73.1)	139 (62.3)	0.104
Operative time, min	36.9 ± 18.2	36.8 ± 20.3	0.969
Anesthesia time, min	41.5 ± 18.4	41.8 ± 20.0	0.921
Extubation time, min	3.5 ± 2.7	3.5 ± 2.9	0.891
Deep extubation	61 (91.0)	196 (87.9)	0.476
Emergence agitation	5 (7.5)	25 (11.2)	0.377
Vomiting	3 (4.5)	16 (7.2)	0.579
Fever	1 (1.5)	5 (2.2)	>0.999
RAEs during extubation to transport	41 (61.2)	58 (26.0)	<0.001*

RAEs, respiratory adverse events; ASA, American society of anesthesiology; BMI, body mass index; VSD, ventricular septal defects; ASD, atrial septal defects; PDA, patent ductus arteriosus; URI, upper respiratory tract infection.

^a^ Continuous data are shown as mean ± standard deviation and categoric data as number (%).

* Statistically significant (*P* < 0.05).

### Model performance and internal validation

**Figure 4:**
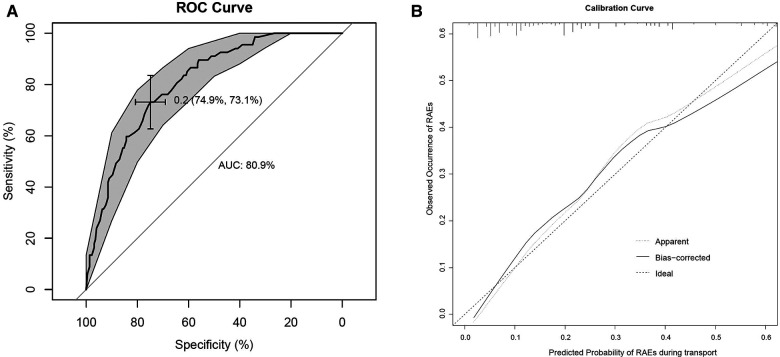
(**A**) AUROC curve for RAEs prediction model. (**B**) Internal calibration curves. A completely accurate prediction model will generate a plot where the probability of the actual observed and predicted corresponding completely, and fall along the 45° line (dashed line). The apparent calibration curve (dotted line) represents the calibration of the model in the development data set, while the bias-corrected curve (solid line) is the calibration result after correcting the optimism with the 1,000 bootstrap-resampling.

H-L goodness-of-fit test value was 0.844. The C- statistic value of the prediction model was 0.809 (95% CI, 0.755–0.862, *P* < 0.001), which showed good discrimination. The sensitivity and specificity based on AUROC curve were 73.1% and 74.9%, respectively (Figure 4A). The apparent calibration curve was close to the 45° ideal line, indicating that the observed probability was consistent with predicted probability in the development cohort (Figure 4B). To lessen the optimism of the model, internal validation with 1,000 bootstrap approach was conducted, which reflect good discrimination with optimism-corrected C- statistic of 0.782 (95% CI, 0.726–0.837). And the bias-corrected calibration curve also demonstrated that the prediction model was well calibrated when the actual observed probability of RAEs during transport was less than 40% (Figure 4B).

### DCA for the development prediction model

**Figure 5:**
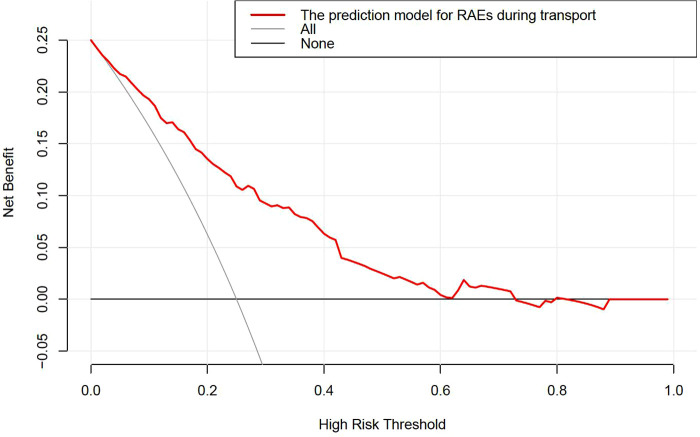
The DCA shows the clinical usefulness of the nomogram. The *Y*-axis represents net benefit. The solid red line is a nomogram predicting the risk of RAEs during transport. The solid gray line indicates that all patients occurred RAEs during transport, while the fine solid black line indicates that no patient occurred RAEs during transport. This DCA could provide a larger net benefit, with ranges of 0%–74%. DCA, decision curve analysis; RAEs, respiratory adverse events.

The depicted DCA was used to determine whether decisions based on the predictive model had clinical applicability compared to the default strategy. Such analyses provide insight into the range of predicted risk for which the model has a high net benefit than simply either treating all (slope line) patients versus treating no (horizontal line) patient, that is to say, a prediction model is only useful at the threshold risk. The depicted DCA indicated the expected net benefit (red curve) per patient for predicting the risk of RAEs during transport. Within the threshold risk range of 0%–74%, intervention decisions based on the predictive model are clearly beneficial (Figure 5).

## Discussion

The incidence of RAEs during transport after interventional cardiac catheterization in pediatric patients was 23.1%. This study identified six independent predictors for RAEs during transport, of which morphine equivalent and atropine usage were modifiable factors that could be optimized to reduce the occurrence of RAEs. Using these six parameters, this study constructed a nomogram to estimate the risk of RAEs during transport, with good C-statistic and calibration in internal validation. The DCA also indicated the clinical usefulness of the nomogram, namely, intervention decisions based on the predictive model were clearly beneficial when the threshold risk range of 0%–74%.

Our study addresses an important knowledge gap in the medical literature regarding the incidence and predictors of RAEs during transport after this surgical procedure in pediatric patients. Previous scholars mainly focused on the construction of different prediction models for RAEs in the period of anesthesia induction, PACU or perioperative, and most of them were retrospective nature with insufficient efficacy (3, 6–10). Our findings suggest that high rate of RAEs during transport deserves our sufficient attention and medical care in the context of the relative lack of medical resources. The prediction model constructed based on a prospectively collected data can effectively predict the risk of RAEs during transport, which is helpful for the identification of high-risk groups and the adjustment of transport plans. Importantly, as two adjustable factors, morphine equivalent and atropine usage have important clinical implications for guiding clinicians to formulate feasible schemes to further reduce the occurrence of RAEs.

Previous documents have identified several underlying predictors for RAEs in pediatric patients during perioperative period, including younger age, ASA grade, race, obesity, obstructive sleep apnea, preexisting pulmonary disorder, URI, premedication, passive smoking, anesthetic technique, anesthetic care without a pediatric anesthetist, type of surgery, and operative time (6–10, 19, 23). By comparison, this study also demonstrated that age ≤3 years, preoperative URI, type of surgery, morphine equivalent and atropine usage, and RAEs during extubation to transport were independent predictors for RAEs during transport. Among these factors, morphine equivalent and atropine usage were rarely reported in the literature.

An important finding of this study is that morphine equivalent and atropine usage are two important modifiable factors that can reduce the risk of RAEs during transport. Opioid dose is significantly positively correlated with perioperative adverse outcomes and long-term prognosis (22, 25). However, there is no literature reporting the effect of opioid dosage on RAEs during pediatric anesthesia. In order to more accurately assess this potential effect, we standardized opioids commonly used in clinical practice, such as fentanyl and sufentanil, according to the analgesic conversion ratio and calculated the dosage under normalized lean body weight, which has clinical practicality (20–22). Our findings echoed the understanding of previous studies that high doses of opioid usage per kg were associated with an increased risk of RAEs during transport. Due to its unique pharmacokinetics and association with postoperative hyperalgesia, remifentanil dose was not included in the opioid calculations but was adjusted as *a priori* defined covariate in the regression models.

Atropine usage, the other of the two adjustable variables, was associated with a lower incidence of RAEs during transport, providing a new insight into medication usage. The underlying biological mechanism is that atropine usage can reduce the production of airway secretions, thereby reducing the risk of RAEs. In the previous literature, the premedication used to prevent or minimize RAEs mainly includes sedative drugs and local anesthetics, such as dexmedetomidine, midazolam, and lidocaine topicalization of the airway (3, 7, 26–28). In our published study, we have confirmed that premedication with intranasal dexmedetomidine was an effective method to decrease the occurrence of RAEs in children with CHD (28). It has also been proven to be beneficial in pediatric patients receiving tonsillectomy and adenoidectomy (3). However, there are conflicting studies of midazolam and RAEs. A national cohort study showed that midazolam usage has a preventive effect on RAEs (29), whereas several studies found that premedication with midazolam appears to increase the incidence of RAEs (3, 7).

In terms of model construction, this study was the first to predict RAEs during transport after this pediatric surgery, and all variables inserted in the predictive model were quantifiable predictors readily available to the clinicians. Besides, the nomogram can provide a visual point system to estimate the probability of RAEs during transport with good discrimination after internal validation. Bias-corrected calibration curve showed that the model could accurately predict the occurrence of RAEs during transport when the observed probability of RAEs during transport was less than 40%. Inversely, a few existing models that cannot accurately and objectively predict RAEs during anesthesia induction or perioperative period, and do not include a special type of surgery such as interventional cardiac catheterization (8, 30–32). Based on the clinical data of 19,095 pediatric patients undergoing elective ambulatory anesthesia for surgery and radiology, Subramanyam et al. developed and validated a risk prediction model for the occurrence of RAEs from the onset of anesthesia induction until discharge from the PACU, with a C-statistic of 0.64 (8).

## Strengths and limitations

Our study has several important strengths. This was an observational study based on a prospectively collected database, and the statistical methods and main outcomes were developed and completed before the start of this trial. To the best of our knowledge, this study was the first to investigate the incidence and predictors, and to construct a nomogram for accurately predicting the occurrence of RAEs during transport after this pediatric surgical treatment. Likewise, several limitations are among our research. First, as a monocentric cohort study, it has the inherent design biases. Second, for the specific surgical type of interventional cardiac catheterization, the pace and the limited time available for postoperative recovery and transport in pediatric patients may slightly increase the occurrence of RAEs during transport. Third, although the prediction model had good performance in internal validation, external validation in a multicenter setting was still required. Finally, randomized controlled trials are needed to confirm whether the two newly reported modifiable factors, morphine equivalents and atropine usage, have an effect on RAEs during transport or even during perioperative period.

## Conclusion

This prospective study explored the incidence and predictors, and constructed a novel nomogram for predicting the occurrence of RAEs during transport. The high rate of RAEs during transport after this pediatric surgical procedure reminds us of the need for more medical care and attention. Six independent predictors for RAEs during transport were identified, of which morphine equivalents and atropine usage were newly reported. Using these six parameters, this study established a novel nomogram, which can reliably identify pediatric patients at high risk of RAEs during transport and guide clinicians to make proper transport plans. Our findings have important and meaningful implications for RAEs risk prediction, clinical intervention and healthcare quality control.

## Data Availability

The original contributions presented in the study are included in the article/Supplementary Material, further inquiries can be directed to the corresponding author/s.

## References

[1] Murat I Constant I Maud'huy H. Perioperative anesthetic morbidity in children: a database of 24,165 anesthetics over a 30-month period. Paediatr Anaesth. (2004) 14(2):158–66. 10.1111/j.1460-9592.2004.01167.x 14962332

[2] von Ungern-Sternberg BS Sommerfield D Slevin L Drake-Brockman TFE Zhang G Hall GL. Effect of albuterol premedication vs placebo on the occurrence of respiratory adverse events in children undergoing tonsillectomies: the REACT randomized clinical trial. JAMA Pediatr. (2019) 173(6):527–33. 10.1001/jamapediatrics.2019.0788 31009034 PMC6547220

[3] Shen F Zhang Q Xu Y Wang X Xia J Chen C Effect of intranasal dexmedetomidine or midazolam for premedication on the occurrence of respiratory adverse events in children undergoing tonsillectomy and adenoidectomy: a randomized clinical trial. JAMA Netw Open. (2022) 5(8):e2225473. 10.1001/jamanetworkopen.2022.25473 35943745 PMC9364121

[4] Tay CL Tan GM Ng SB. Critical incidents in pediatric anesthesia: an audit of 10 000 anesthetics in Singapore. Pediatr Anaesth. (2001) 11(6):711–8. 10.1046/j.1460-9592.2001.00767.x 11696149

[5] Oofuvong M Geater AF Chongsuvivatwong V Chanchayanon T Sriyanaluk B Saefung B Excess costs and length of hospital stay attributable to perioperative respiratory events in children. Anesth Analg. (2015) 120(2):411–9. 10.1213/ANE.0000000000000557 25517194

[6] Malviya S Voepel-Lewis T Siewert M Pandit UA Riegger LQ Tait AR. Risk factors for adverse postoperative outcomes in children presenting for cardiac surgery with upper respiratory tract infections. Anesthesiology. (2003) 98(3):628–32. 10.1097/00000542-200303000-00009 12606905

[7] von Ungern-Sternberg BS Boda K Chambers NA Rebmann C Johnson C Sly PD Risk assessment for respiratory complications in pediatric anesthesia: a prospective cohort study. Lancet. (2010) 376(9743):773–83. 10.1016/S0140-6736(10)61193-2 20816545

[8] Subramanyam R Yeramaneni S Hossain MM Anneken AM Varughese AM. Perioperative respiratory adverse events in pediatric ambulatory anesthesia: development and validation of a risk prediction tool. Anesth Analg. (2016) 122(5):1578–85. 10.1213/ANE.0000000000001216 27101501

[9] Tariq S Syed M Martin T Zhang X Schmitz M. Rates of perioperative respiratory adverse events among Caucasian and African American children undergoing general anesthesia. Anesth Analg. (2018) 127(1):181–7. 10.1213/ANE.0000000000003430 29750690

[10] Wudineh DM Berhe YW Chekol WB Adane H Workie MM. Perioperative respiratory adverse events among pediatric surgical patients in university hospitals in northwest Ethiopia; A prospective observational study. Front Pediatr. (2022) 10:827663. 10.3389/fped.2022.827663 35223702 PMC8873930

[11] Lerman J. Perioperative respiratory complications in children. Lancet. (2010) 376(9743):745–6. 10.1016/S0140-6736(10)61199-3 20816527

[12] Tait AR Voepel-Lewis T Christensen R O'Brien LM. The STBUR questionnaire for predicting perioperative respiratory adverse events in children at risk for sleep-disordered breathing. Paediatr Anaesth. (2013) 23(6):510–6. 10.1111/pan.12155 23551934 PMC3648590

[13] Welliver RC Sr Checchia PA Bauman JH Fernandes AW Mahadevia PJ Hall CB. Fatality rates in published reports of RSV hospitalizations among high-risk and otherwise healthy children. Curr Med Res Opin. (2010) 26(9):2175–81. 10.1185/03007995.2010.505126 20666690

[14] Delgado-Corcoran C Witte MK Ampofo K Castillo R Bodily S Bratton SL. The impact of human rhinovirus infection in pediatric patients undergoing heart surgery. Pediatr Cardiol. (2014) 35(8):1387–94. 10.1007/s00246-014-0941-3 24939564

[15] Moynihan K Barlow A Alphonso N Anderson B Johnson J Nourse C Impact of viral respiratory pathogens on outcomes after pediatric cardiac surgery. Pediatr Crit Care Med. (2017) 18(3):219–27. 10.1097/PCC.0000000000001083 28114162

[16] Promphan W Qureshi SA. What interventional cardiologists are still leaving to the surgeons? Front Pediatr. (2016) 4:59. 10.3389/fped.2016.00059 27379218 PMC4904017

[17] Lin CH Hegde S Marshall AC Porras D Gauvreau K Balzer DT Incidence and management of life-threatening adverse events during cardiac catheterization for congenital heart disease. Pediatr Cardiol. (2014) 35(1):140–8. 10.1007/s00246-013-0752-y 23900744 PMC3882522

[18] Zhang S Ding S Cai M Bai J Zhang M Huang Y Impact of upper respiratory tract infections on perioperative outcomes of children undergoing therapeutic cardiac catheterisation. Acta Anaesthesiol Scand. (2018) 62(7):915–23. 10.1111/aas.13113 29569250

[19] Zhang K Wang S Li M Wu C Sun L Zhang S Anesthesia timing for children undergoing therapeutic cardiac catheterization after upper respiratory infection: a prospective observational study. Minerva Anestesiol. (2020) 86(8):835–43. 10.23736/S0375-9393.20.14293-7 32251574

[20] Gordon DB Stevenson KK Griffie J Muchka S Rapp C Ford-Roberts K. Opioid equianalgesic calculations. J Palliat Med. (1999) 2(2):209–18. 10.1089/jpm.1999.2.209 15859817

[21] Ingrande J Lemmens HJ. Dose adjustment of anaesthetics in the morbidly obese. Br J Anaesth. (2010) 105(Suppl 1):i16–23. 10.1093/bja/aeq312 21148651

[22] Long DR Lihn AL Friedrich S Scheffenbichler FT Safavi KC Burns SM Association between intraoperative opioid administration and 30-day readmission: a pre-specified analysis of registry data from a healthcare network in new England. Br J Anaesth. (2018) 120(5):1090–102. 10.1016/j.bja.2017.12.044 29661386

[23] Tait AR Malviya S Voepel-Lewis T Munro HM Seiwert M Pandit UA. Risk factors for perioperative adverse respiratory events in children with upper respiratory tract infections. Anesthesiology. (2001) 95(2):299–306. 10.1097/00000542-200108000-00008 11506098

[24] Van Calster B Wynants L Verbeek JFM Verbakel JY Christodoulou E Vickers AJ Reporting and interpreting decision curve analysis: a guide for investigators. Eur Urol. (2018) 74(6):796–804. 10.1016/j.eururo.2018.08.038 30241973 PMC6261531

[25] Cron DC Englesbe MJ Bolton CJ Joseph MT Carrier KL Moser SE Preoperative opioid use is independently associated with increased costs and worse outcomes after major abdominal surgery. Ann Surg. (2017) 265(4):695–701. 10.1097/SLA.0000000000001901 27429021

[26] Li LW He L Ai Y Chu Q Zhang W. Site-directed topical lidocaine spray attenuates perioperative respiratory adverse events in children undergoing elective surgery. J Surg Res. (2016) 203(1):206–10. 10.1016/j.jss.2016.03.011 27338551

[27] Poonai N Spohn J Vandermeer B Ali S Bhatt M Hendrikx S Intranasal dexmedetomidine for procedural distress in children: a systematic review. Pediatrics. (2020) 145(1):e20191623. 10.1542/peds.2019-1623 31862730

[28] Zhang S Zhang R Cai M Zhang K Zhang M Zheng J. Intranasal dexmedetomidine premedication in children with recent upper respiratory tract infection undergoing interventional cardiac catheterisation: a randomised controlled trial. Eur J Anaesthesiol. (2020) 37(2):85–90. 10.1097/EJA.0000000000001097 31644515

[29] Michel F Vacher T Julien-Marsollier F Dadure C Aubineau JV Lejus C Peri-operative respiratory adverse events in children with upper respiratory tract infections allowed to proceed with anaesthesia: a French national cohort study. Eur J Anaesthesiol. (2018) 35(12):919–28. 10.1097/EJA.0000000000000875 30124501

[30] Lee LK Bernardo MKL Grogan TR Elashoff DA Ren WHP. Perioperative respiratory adverse event risk assessment in children with upper respiratory tract infection: validation of the COLDS score. Paediatr Anaesth. (2018) 28(11):1007–14. 10.1111/pan.13491 30281195

[31] Tao S Zhang T Wang K Xie F Ni L Mei Z Identification of the risk factors in perioperative respiratory adverse events in children under general anesthesia and the development of a predictive model. Transl Pediatr. (2021) 10(7):1877–82. 10.21037/tp-21-257 34430435 PMC8349961

[32] Zhang Q Shen F Wei Q Liu H Li B Zhang Q Development and validation of a risk nomogram model for perioperative respiratory adverse events in children undergoing airway surgery: an observational prospective cohort study. Risk Manag Healthc Policy. (2022) 15:1–12. 10.2147/RMHP.S347401 35023976 PMC8747787

