# Supplementary material for: A novel nomogram for predicting respiratory adverse events during transport after interventional cardiac catheterization in children
Source: Front Pediatr. 2022 Oct 20;10:1044791. doi: 10.3389/fped.2022.1044791 (PMC9631021; doi:10.3389/fped.2022.1044791)
Supplement: Supplementary file 2 [file DataSheet1.pdf]

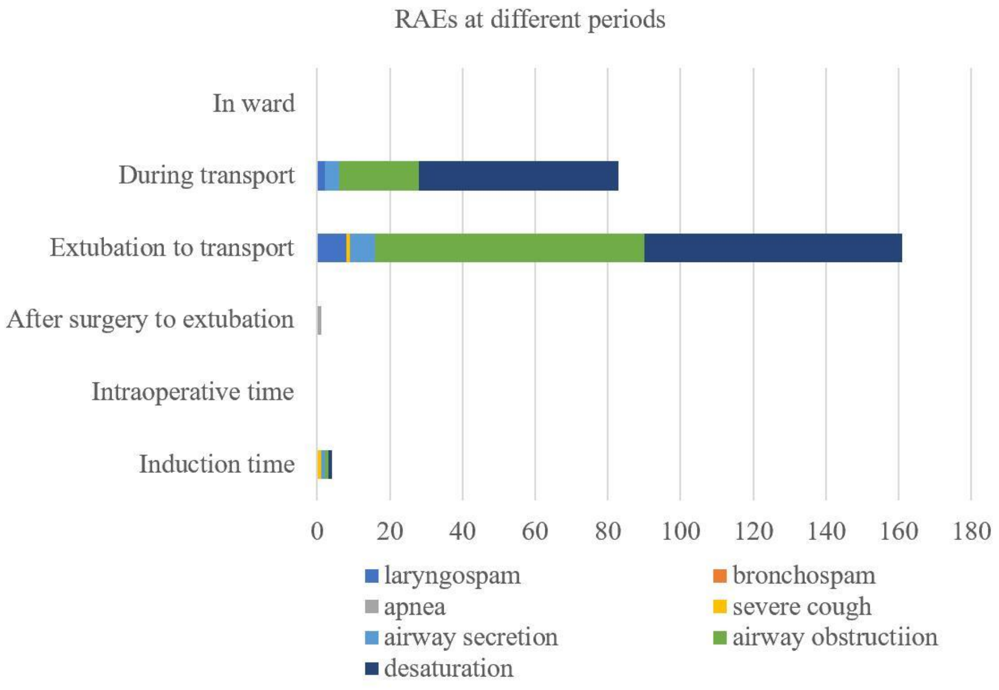

Supplementary Figure S1 RAEs at different periods

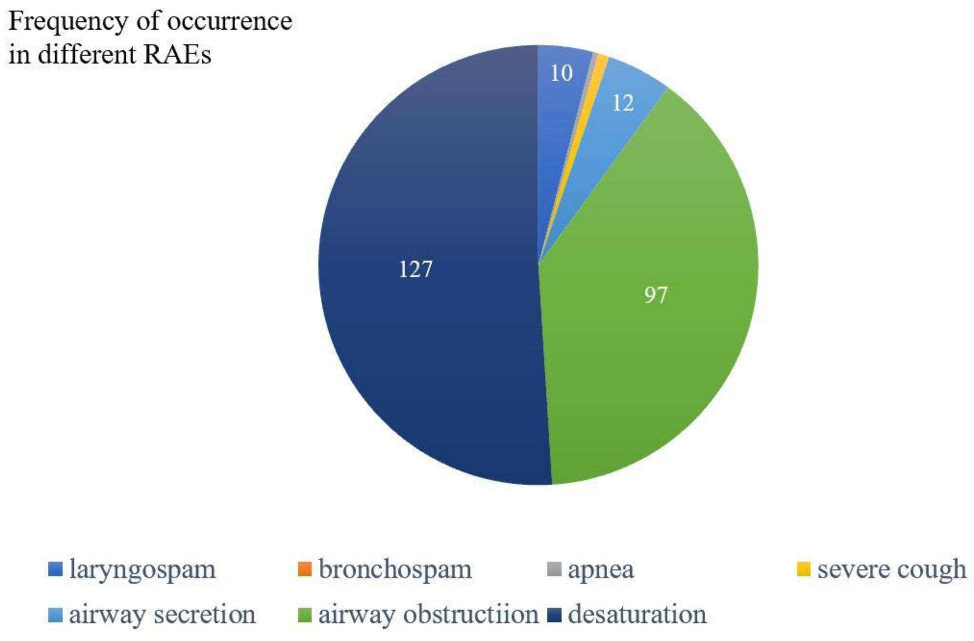

Supplementary Figure S2 Frequency of occurrence in different RAEs
